# Absolute quantification of DcR3 and GDF15 from human serum by LC-ESI MS

**DOI:** 10.1111/jcmm.12540

**Published:** 2015-03-30

**Authors:** Ioana Lancrajan, Regine Schneider-Stock, Elisabeth Naschberger, Vera S Schellerer, Michael Stürzl, Ralf Enz

**Affiliations:** a Institute of Biochemistry (Emil-Fischer-Centre), Friedrich-Alexander-University of Erlangen-NurembergErlangen, Germany; b Experimental Tumorpathology, Institute of Pathology, Friedrich-Alexander-University of Erlangen-NurembergErlangen, Germany; c Division of Molecular and Experimental Surgery, University Medical Centre ErlangenErlangen, Germany; d Department of Surgery, University Medical Centre Erlangen91054, Erlangen

**Keywords:** absolute quantification, serum, biomarker, low-abundance proteins, partial denaturation, mass spectrometry, LC-ESI MS, proteomics

## Abstract

Biomarkers are widely used in clinical diagnosis, prognosis and therapy monitoring. Here, we developed a protocol for the efficient and selective enrichment of small and low concentrated biomarkers from human serum, involving a 95% effective depletion of high-abundant serum proteins by partial denaturation and enrichment of low-abundant biomarkers by size exclusion chromatography. The recovery of low-abundance biomarkers was above 97%. Using this protocol, we quantified the tumour markers DcR3 and growth/differentiation factor (GDF)15 from 100 μl human serum by isotope dilution mass spectrometry, using ^15^N metabolically labelled and concatamerized fingerprint peptides for the both proteins. Analysis of three different fingerprint peptides for each protein by liquid chromatography electrospray ionization mass spectrometry resulted in comparable concentrations in three healthy human serum samples (DcR3: 27.23 ± 2.49 fmol/ml; GDF15: 98.11 ± 0.49 fmol/ml). In contrast, serum levels were significantly elevated in tumour patients for DcR3 (116.94 ± 57.37 fmol/ml) and GDF15 (164.44 ± 79.31 fmol/ml). Obtained data were in good agreement with ELISA and qPCR measurements, as well as with literature data. In summary, our protocol allows the reliable quantification of biomarkers, shows a higher resolution at low biomarker concentrations than antibody-based strategies, and offers the possibility of multiplexing. Our proof-of-principle studies in patient sera encourage the future analysis of the prognostic value of DcR3 and GDF15 for colon cancer patients in larger patient cohorts.

## Introduction

Human serum is a complex biological fluid widely used for clinical diagnosis, determination of prognosis and monitoring of therapy. Diseased organs may leak proteins into the blood stream that can be detected and correlated to pathophysiological conditions in patients. Thus, proteins that change their concentration, their isoform or post-translational modification in sera of patients in a disease-related manner may be useful biomarkers 1. Therefore, it is not surprising that the identification and quantification of biomarkers is of high interest and represents a competitive field of research. Today, biomarkers are known for several diseases, including cancer, multiple sclerosis, hepatitis and schizophrenia 2.

The protein composition in human serum is extremely diverse, containing several thousand different protein species, of which most are of low abundance. The concentration range of serum proteins covers a dynamic range of about ten orders of magnitude, from several mg to some pg per ml. In contrast, typical analytical techniques such as liquid chromatography (LC) or mass spectrometry (MS) are able to detect proteins in the range 2 to 4 orders of magnitudes, only 3. Furthermore, the most abundant protein albumin represents about 50% of the total protein content of human serum and the 12 highest concentrated proteins together comprise more than 95% of all proteins present 4. In contrast, clinically relevant biomarkers are generally low concentrated and therefore difficult to detect. Therefore, identification and quantification of low-abundance serum proteins is not trivial.

Especially the reduction in the diversity of serum proteins is a critical step for the detection of biomarkers. Compared to high concentrated serum proteins, such as albumin and immunoglobulins, the majority of low-abundance proteins has also a low molecular weight. Thus, besides the use of specific antibodies, also this size difference is used to enrich biomarkers from serum. Common strategies that separate high-abundant proteins from low-abundant biomarkers are centrifugal ultrafiltration, thermal treatment, microarray techniques, two-dimensional gel electrophoresis, organic solvent extraction, affinity chromatography, LC, the protein equalizer technology and the use of combinatorial ligand libraries 2,3,5–7. In most cases, a combination of techniques is used. Recently, more unconventional techniques to purify and enrich serum proteins prior to MS detection were described, including the use of nanoporous silicon microparticles or mesoporous silica chips 8,9.

High-abundance proteins, such as albumin and immunoglobulins can serve as a sponge for low-abundant proteins in the blood. It has been estimated that albumin and immunoglobulins bind 20% or 48% of plasma proteins 3. Depleting high-abundance proteins from human serum therefore carries the risk to co-deplete and thereby loose low-abundant biomarker proteins. As a consequence, some groups took the opposite approach and purified high-abundance proteins with subsequent analysis of their protein content 5. In addition, there is evidence that tumour-related exoproteases cleave high-abundance plasma proteins, which results in so-called ‘peptide ladders’ that are detectable by MS techniques 10,11.

Despite numerous efforts to identify reliable biomarkers, *e.g*. for tumour diagnosis only a limited number of biomarkers are currently used in the clinic, including alpha-fetoprotein, carcinoembryonic antigen (CEA), prostate-specific antigen or the cancer antigens 15-3, 19-9 and 125 12. During the last years, most prominent techniques for the analysis of biomarkers in cancer patients include antibody-based methods, such as ELISA that have established the use of CEA 13, death decoy receptor 3 (DcR3) and Spondin 2 14 and growth/differentiation factor 15 (GDF15) 15 as reliable tumour markers. In colorectal cancer, one of the most common malignancies worldwide, several serum proteins are discussed as promising biomarkers, including DcR3 and GDF15 16.

The biggest drawback of antibody-based methods is the need of specific, highly affine antibodies for each biomarker. Mass spectrometry represents an alternative, fast and reliable detection technique that has been used to detect a large variety of biomarkers, including endogenous metabolites such as cholesterol derivates (androgens, 4β-hydroxycolesterol), or peptides and proteins and their post-translational modifications 17–20. Here, we present a new method based on partial denaturation of serum proteins by organic solvents, combined with size exclusion chromatography and followed by the absolute quantification of serum proteins by liquid chromatography electrospray ionization mass spectrometry (LC-ESI MS), using isotope labelled and concatamerized fingerprint peptides.

## Materials and methods

### Patient characteristics

Sera samples were obtained from patients undergoing standard surgical procedure for primarily diagnosed with colorectal cancer at the Department of Surgery. Tumours were histopathologically characterized according to the Union International Contre le Cancer. A collective of 19 patients with different histopathological grading (12 samples for G_1_/G_2_ and seven samples for G_3_/G_4_) was selected for biomarker quantification. While our studies were on-going, one patient (G_3_) had no tumour resection and thus his sample was excluded. The clinicopathological characteristics of the patients are given in Table S1. The procedure was approved by the local Ethics Committee and all patients provided written informed consent.

### Denaturation and trypsin digestion of human serum proteins

To investigate the impact of partial protein denaturation on trypsin proteolysis, 10 μl normal goat serum (Dianova, Hamburg, Germany) was diluted in H_2_O, and mixed with methanol or acetonitrile (AcCN) to final concentrations between 40% and 60% in a total volume of 200 μl. Thereafter, each sample was treated with 50 μl trypsin (20 μg trypsin from porcine pancreas proteomics grade was reconstituted in 500 μl 28 mM ammonium bicarbonate buffer including 10% AcCN according to the manufacturers’ protocol; Sigma-Aldrich, Taufkirchen, Germany) and incubated for 1, 18, 24 or 48 hrs at 37°C. After 24 hrs, additional 25 μl trypsin was added. The reaction volume of 15 μl was incubated with loading buffer without DTT (125 mM Tris/HCl pH 6.8, 4% SDS, 20% glycerol) for 10 min. at 95°C. Proteins were separated on SDS-PAGE and visualized by Commassie blue staining.

To analyse the effect of AcCN denaturation in more detail, 10 μl of human serum prepared from normal male type AB plasma samples (S2145, H2257 or H4522 from Sigma-Aldrich) was diluted in different solutions (H_2_O, 50 mM phosphate buffer pH 7.5 or PBS pH 7.5) and mixed with AcCN to final concentrations between 0% and 80% in a total volume of 200 μl. After incubation at 20°C for 1 hr, samples were centrifuged at 12,000 × g for 10 min. The supernatant was separated from the precipitate and vacuum dried (Concentrator 5301, Eppendorf, Hamburg, Germany). Both, precipitates and dried supernatants were resuspended in 200 μl of their corresponding solutions described above. For each AcCN concentration tested, 15 μl of supernatant and of resuspended sediments was loaded on SDS-PAGE and proteins were visualized as described above. To identify low-abundance proteins, 100 μl of the above described resuspended precipitates and dried supernatants of AcCN-treated human serum samples were analysed by dot blots on Hybond-PVDF membranes (GE Healthcare, Freiburg, Germany), using a polyclonal immuneserum raised in rabbits directed against several low-abundant proteins that are described in the literature as promising tumour markers in colorectal cancer, including DcR3, GDF15, M2-PK, PSME3 and TIMP-1 16 (1:100; the immuneserum was generated by Davids-Biotechnologie GmbH, Regensburg, Germany and kindly provided by Markus Fischer, Entelechon GmbH, Bad Abbach, Germany). Proteins were visualized with a mouse monoclonal secondary antibody (1:10,000; peroxidase-conjugated IgG light chain fraction; Dianova) and the SuperSignal West Pico Chemiluminescent Substrate (Thermo Fisher Scientific, Bonn, Germany).

To estimate the recovery rate of low-abundance proteins, 10 μg porcine trypsin (Sigma-Aldrich) was treated with 60% AcCN in H_2_O as above, remaining protein in the soluble fraction was analysed by SDS-PAGE and signal intensities of untreated and AcCN-treated trypsin were compared with ImageLab 2.0.1 (Bio-Rad, München, Germany). Immunodepletion of human serum albumin was performed with 10 μl serum and the Vivapure anti-HSA kit (Sartorius Stedim Biotech GmbH; Göttingen, Germany) following the manufacturers’ protocol. Protein concentrations were measured with the BCA Protein Assay Kit (Thermo Fisher Scientific).

### Size exclusion chromatography

For later analysis by MS, the above described protocol of human serum protein denaturation in the presence 60% AcCN was scaled up by a factor of 10. One hundred μl of human serum was diluted 1:1 (v/v) with H_2_O and 300 μl AcCN to achieve a final concentration of 60%. Thereafter, the solution was sonicated for 10 min. in a ultrasonic bath at 120 Watt at 20°C (Bandelin Sononrex; Schalltec GmbH, Mörfelden-Walldorf, Germany) and centrifuged at 12,000 × g for 10 min. For chromatographic purification, the supernatant was vacuum dried as above, resuspended in 100 μl H_2_O and injected in a gel filtration column (Superdex 75 10/300 GL; GE Healthcare) in a HPLC (LaChrom Elite, Hitachi, Schaumburg, IL, USA) with 5 mM phosphate buffer (pH 7.4) containing 150 mM NaCl as solvent and a flow rate of 0.5 ml/min. Thirty fractions of 1.5 ml were collected, each fraction containing about 1.2 μg protein. Fractions 9 to 24 were vacuum dried and resuspended in 180 μl H_2_O. The column was calibrated with a gel filtration standard kit (Bio-Rad) according to the manufacturer’s instructions.

### MALDI-TOF MS

To prepare the 180 μl volume containing chromatographic protein fractions for analysis by MALDI-TOF MS, disulphide bonds were reduced by the addition of 10 μl 100 mM dithiothreitol (diluted in H_2_O) and an incubation of 1 hr at 60°C. In addition, alkylation of cysteine containing peptides was performed by adding 10 μl 100 mM iodoacetamide (diluted in H_2_O) and an incubation of 60 min. at 20°C in the dark. Each sample was trypsin digested in the presence of 10% AcCN as described above, vacuum dried, resuspended in 20 or 100 μl 0.1% trifluoracetic acid (TFA) and desalted on ZipTip C18 P10 (Millipore, Merck, Darmstadt, Germany) according to the manufacturer’s instructions. Obtained solutions were air dried, and dissolved in 1 μl 0.1% TFA. Thereafter, 1 μl 2% TFA and 1 μl 2′,4′,-dihydroxyacetophenone matrix were added and an aliquot of 0.5 μl was dotted on a steel target (MTP 384; Bruker Daltonics, Bremen, Germany) and allowed to air dry. MALDI-TOF analysis was performed on an Autoflex mass spectrometer (Bruker Daltonics) in the positive reflector mode, using a nitrogen laser (337 nm) for sample desorption and an acceleration with 20 kV after a delay of 3500 nsec. Six individual spectra, each generated by 50 shots/individual spectrum recorded from different positions of a sample spot were used to generate the final mass spectra. Spectra were analysed using Flex Analysis software (Bruker Daltonics).

Masses of labelled and unlabelled peptide sequences representing DcR3 and GDF15 were calculated with the MS Digest software (Protein Prospector at http://prospector.ucsf.edu). The data were transformed into MGF files for database searches with the Mascot® search algorithm (Matrix Science, London, UK, version 2.2.0), using a peptide mass tolerance of 0.3 D. For the protein identification the enzyme settings were set on ‘trypsin’ and up to 1 possible missed cleavage sites were considered for the database searches, as well as the following variable modification: nitro (Y), and oxidation (M). All data were searched against the human SwissProt database.

### Protein expression, metabolic labelling and trypsination of Q1

To generate control peptides for the absolute quantification of low-abundance proteins by isotope dilution mass spectrometry (IDMS), we used the protein construct Q1 in which characteristic peptide sequences that represent different tumour markers were concatamerized and can be separated by trypsination. Q1 was synthesized in E.coli, purified by affinity chromatography using a C-terminal His-tag and kindly provided by Markus Fischer (Entelechon GmbH, Bad Abbach, Germany). To obtain ^15^N isotopically labelled peptide sequences, M9 minimal medium with ^15^NH_4_Cl as sole nitrogen source was used.

Concentrations of synthesized Q1 proteins were measured by Bradford using the BCA protein assay kit (Thermo Fisher Scientific) according to the manufacturer’s protocol. In addition, we measured the Q1 concentration by densitometry. Various amounts of Q1 protein were visualized on a 11% SDS-PAGE by Commassie blue staining and protein concentrations were calculated relative to the BSA standard curve using Molecular Imager ChemiDoc™ XRS with ImageLab™ software (Bio-Rad).

To test the stability of Q1 in freeze/thaw cycles, 1.6 μg aliquots of the purified Q1 protein were subjected to 3 cycles between −20°C and +20°C. To analyse the stability of Q1 at the digestion temperature for trypsin at 37°C, the same amount (1.6 μg) was incubated at 37°C for 24 and 48 hrs. To ensure completeness of proteolysis, other Q1 aliquots (2.4 μg) were incubated at 37°C between 0 and 5 hrs in the absence or presence of trypsin. In all cases, protein degradation was analysed by standard SDS-PAGE and Commassie blue staining.

To analyse the efficiency of ^15^N labelling, 1200 fmol of unlabelled and labelled Q1 were mixed together, digested and analysed by MALDI-TOF, as above. For comparison, spectra of trypsin-digested-unlabelled Q1 were recorded.

### LC–ESI MS

To analyse the visibility of fingerprint peptides, trypsin-digested samples (1200 fmol metabolically labelled and unlabelled Q1 protein) were analysed by LC tandem MS (LC-MS/MS), in which an Esquire6000 ion trap (Bruker Daltonik) was coupled with an Agilent 1200-series binary pump system (Agilent Technologies, Alpharetta, GA, USA). The reverse phase column used was an analytical C18 column (2.1 × 150 mm, 3.5 μm, 300 Å) XBridge (Milford, MA USA). Peptides were eluted from the column with a linear AcCN gradient that was generated from a mixture of 0.1% formic acid in water (solvent A) and 0.1% formic acid in AcCN (solvent B) and consisted of 0% to 70% solvent B for 40 min., followed by 98% solvent B for 85 min., with a flow rate of 0.3 ml/min. at 40°C. Full-scan mass spectra were acquired by ESI in the positive-ion mode over a m/z range of 100 to 3000. The ESI source was operated at 350°C dry gas temperature. Fragmentation was induced using nitrogen at high-pressure settings in auto MS2 modus. The chromatographic peaks representing fingerprint peptides were identified using extracted ion chromatograms (0.3 D mass range over a 4 min. chromatographic window) by plotting the intensity of the signal at the theoretical m/z values recorded in SwissProt using MS Digest Software.

To test sensitivity and linearity of our detection technique, the trypsin-digested size exclusion chromatography eluat fraction 11 or 13 were vacuum dried, resolubilized in 60 μl solvent A and supplemented with 10, 30, 100 or 300 fmol of trypsinated-labelled Q1 protein. ESI spectra were recorded as above, using a linear AcCN gradient consisted 0% to 98% solvent B for 110 min., followed by 98% B for 5 min. and 100% B for 15 min. at 30°C.

For the absolute quantification of DcR3 and GDF15, a defined amount of ^15^N isotopically labelled fingerprint peptides served as internal standard for their respective light counterpart. To generate the fingerprint peptides, 5.8 μg (99.15 pmol) of metabolically labelled and purified Q1 protein (vacuum dried) was mixed with 110 μl distillated water, 40 μl AcCN, 50 μl trypsin (1 μg; Sigma-Aldrich) and incubated at 37°C for 5 hrs. Every hour 25 μl trypsin solution was added to the reaction mixture and after 5 hrs digestion the reaction was stopped. Fifty fmol labelled peptides were mixed with the same volume of trypsin-digested size exclusion chromatography eluat fraction 11 or 13. Thereafter, samples were vacuum dried, dissolved in 60 μl solvent A and analysed by LC–ESI MS as above, using a linear AcCN gradient of 0% to 98% B for 110 min., 98% B for 5 min. and 100% B for 15 min. at 30°C. Serum concentrations of DcR3 and GDF15 were calculated from ratios between peak areas of analyte fingerprint peptide and the peak areas of the corresponding ^15^N-metabolic-labelled Q1 concatamer-derived peptides.

### Enzyme-linked immunosorbent assay

DcR3 and GDF15 levels were analysed by an ELISA kit (DcR3: Ray Biotech, Norcross, GA, USA, calibration range between 9 and 2450 fmol/ml; GDF15: BioVendor, Brno, Czech Republic, calibration range between 6 and 360 fmol/ml) on capture antibody pre-coated microtitre plates. Aliquots of serum (both in 1:5 dilution) were pipetted in triplicate into the wells and incubated for 2.5 hrs (DcR3) or 1 hr (GDF15) at room temperature. After washing, the biotinylated DcR3 or GDF15 antibody was added and incubated for 1 hr at room temperature. After washing, streptavidin-horseradish peroxidase conjugate was added and incubated for 45 min. (DcR3) or 30 min. (GDF15). After washing, a substrate solution was added for colorimetric detection. After stopping the reaction, the optical density was measured in a spectrophotometer (Perkin Elmer, Rodgau, Germany). Calibration curves were made with DcR3 or GDF15 standards provided in the kits. The amounts of DcR3 and GDF15 in the serum samples were determined by extrapolation using calibration curves. Statistical analysis was performed with SPSS version 16.0 (SPSS, Chicago, IL, USA) and Origin (Microcal Software, Northhamton, MA, USA). *P* < 0.05 were considered statistically significant.

### Quantitative real-time RT-PCR

Expression of GDF15 mRNA was analysed by quantitative real-time RT-PCR (qPCR). Briefly, RNA was isolated from one 5 μm tissue slice where tumour and non-tumour regions have been marked (Recover all™, Total nucleic acid isolation, Ambion, LifeTechnologies, Grand Island, NY, USA). cDNA synthesis of 30–250 ng total RNA (miScriptII RT kit; Qiagen, Hilden, Germany) was performed according to the manufacturer’s instruction. Three μl cDNA was amplified in a thermal cycler (Bio-Rad CFX-96) using the QuantiTectSybrgreen Mastermix, Qiagen). PCR conditions were 95°C for 15 min. and 40 cycles of 95°C for 30 sec., 59°C for 30 sec. and 72°C for 60 sec. GDF15-specific primers (Metabion, Steinkirchen, Germany) were forward: 5′-cccatggtgctcattcaaaag-3′ and reverse: 5′-gctcatatgcagtggcagtctt-3′. As housekeeping gene we used the β2-microglobulin (β2M) gene with primers F: 5′-tgactttgtcacagcccaagata-3′ and R: 5′-aatccaaatgcggcatcttc-3′. Results are expressed as 2^(Ctβ2M-CtGDF15)^.

## Results

To quantify low-abundant proteins in human serum, we combined an antibody free method for the depletion of high-abundance proteins with the quantification concatamer (QconCAT) technology, originally introduced by the group of Robert J. Beynon 21,22 and with IDMS. The low-abundant serum proteins DcR3 and GDF15 served as model substrates.

### Precipitation and proteolysis of serum proteins

Ideally, a purification step prior to MS enriches low-abundant biomarkers and ensures their complete proteolysis for a reliable identification *via* mass fingerprints. For the initial development of an efficient purification protocol, goat serum was used. DcR3 and GDF15 are of low abundance and represent rather small proteins of about 30 kD, when compared with the typical high-abundant serum proteins, such as albumin and immunoglobulins. We took advantage of this size difference and depleted high molecular weight proteins from with denaturating solutions containing different organic solvents (acetonitrile – AcCN, methanol – MeOH) in various concentrations (40–60%).

**Figure 1:**
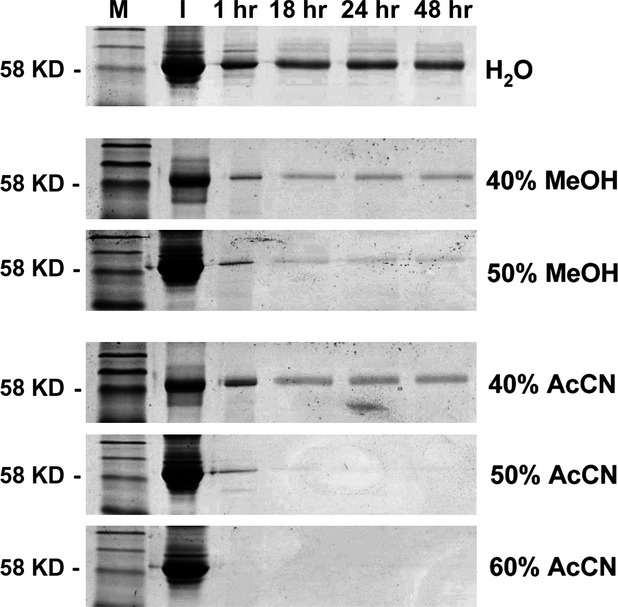
Organic solutions enhance trypsin digestion of serum proteins. Goat serum of 10 μl was digested with trypsin for the indicated hours (h), serum proteins were analysed on SDS-PAGE and visualized by Commassie staining. While proteolysis without addition of organic solutions was incomplete after 48 hrs (upper panel), partial denaturation of serum proteins by the addition of methanol (MeOH) or acetonitrile (AcCN) to the reaction mixture significantly increased proteolysis efficiency. A concentration of 50–60% AcCN in the digestion mixture resulted in a complete proteolysis of serum proteins within 18 hrs or 1 hr respectively. (I – Input protein amount; M – protein marker in kD).

In addition to the depletion of large serum proteins, partial denaturation has the potential to partially unfold smaller proteins, thereby increasing their trypsin digestibility. First, we monitored the amount of proteolysis by SDS-PAGE with respect to the most prominent proteins visible in the Commassie-stained gels at a molecular mass of around 60 kD. Generally, most protein was digested within the first hour of trypsin incubation (Fig.1). However, even after 48 hrs the proteolysis of serum proteins was not complete (Fig.1, upper panel). Addition of 40% or 50% methanol improved protein digestion, but the amount of denaturation was not sufficient for a complete proteolysis within 48 hrs. In contrast, digestion mixtures containing 50% or 60% AcCN resulted in a total proteolysis of human serum proteins after 1 or 18 hrs (Fig.1).

**Figure 2:**
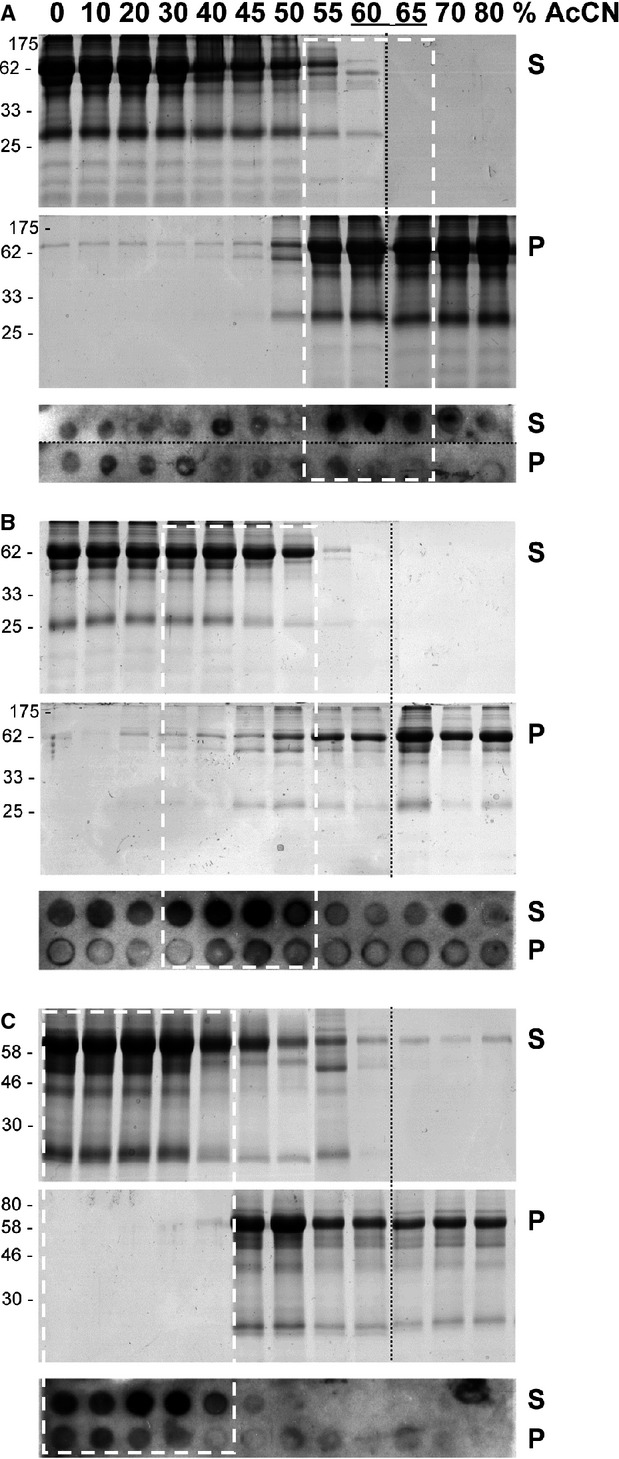
Partial denaturation of serum proteins by acetonitrile at different salt concentrations. Human serum of 10 μl was diluted in H_2_O (A), 50 mM phosphate buffer pH 7.5 (B), or phosphate buffer saline (pH 7.5) (C). For partial denaturation of serum proteins, various volumes of acetonitrile (AcCN) were added to final concentrations between 0% and 80%. A centrifugation step generated soluble (S) and precipitated (P) proteins that were analysed on Commassie-stained SDS-PAGE to monitor the behaviour of high-abundance proteins (upper panels), or by dot blot analysis (lower panels) using an immuneserum directed against low-abundant tumour markers. White rectangles indicate conditions under which low-abundant proteins detected on the dot blot remain in the soluble fraction. Only at AcCN concentrations of 60% and 65% in H_2_O (underlined lanes in A), most high-abundant proteins visible in the Commassie-stained SDS-PAGE were depleted from the soluble fraction, whereas low-abundant proteins remain soluble. Dotted black lines indicate assembly of different protein gels or dot blot membranes into one panel for clearer data representation. Protein marker sizes are indicated in kD on the left.

As addition of AcCN seemed to be most promising, we next analysed the behaviour of human serum proteins in the presence of various AcCN concentrations in different solutions representing increasing salt concentrations from 0 to about 140 mM (H_2_O, 50 mM sodium phosphate buffer pH 7.5 or PBS pH 7.5; Fig.2). The solubility of high-abundance serum proteins after AcCN fractionation was monitored after a centrifugation step on Commassie-stained SDS-PAGE. In all three solutions tested, serum proteins disappear from the supernatant and become visible in the precipitate at AcCN concentration between 50% and 60%, indicating that most high-abundant proteins were efficiently denatured and precipitated under these conditions. To analyse the behaviour of low-abundant serum proteins, we stained the same protein fractions on a dot blot with a polyclonal immuneserum directed against proteins that are described in the literature as promising tumour markers in colorectal cancer, including DcR3 and GDF15 16. The dot blots show that the presence of soluble low-abundant serum proteins shifts to lower AcCN concentrations with increasing salt concentration (white boxes in Fig.2). Only at AcCN concentrations of 60% to 65% in H_2_O most of the smaller human serum proteins remained in the soluble fraction, whereas under the same conditions most high molecular weight proteins were precipitated (Fig.2A, underlined lanes).

**Figure 3:**
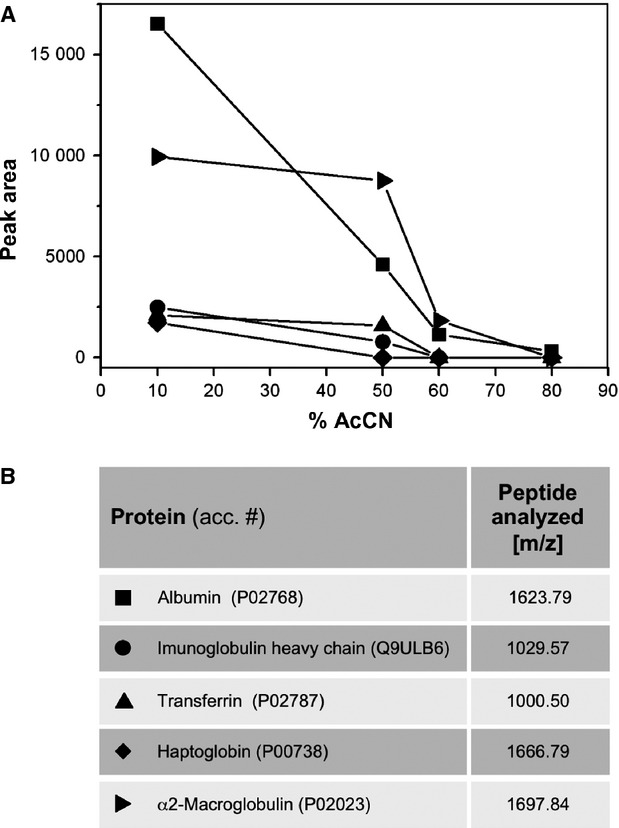
Depletion of high-abundance proteins by acetonitrile. Human serum of 10 μl was diluted in H_2_O and incubated in the presence of 10%, 50%, 60% or 80% acetonitrile (AcCN) (A). After centrifugation, soluble proteins in the supernatant were trypsin digested for 48 hrs and resulting peptides were analysed by MALDI-TOF MS (B). For selected high-abundance proteins, the area under representative mass peaks was measured and plotted against the used AcCN concentration, as indicated. An efficient depletion of most high-abundance proteins at 60% AcCN is evident (m/z – mass to charge ratio of mono isotopic masses).

To evaluate the efficacy of the described procedure in more detail, we monitored (*i*) the loss of specific peptide signals representing high-abundance proteins during the precipitation procedure, (*ii*) the overall amount of depletion of high-abundance proteins and (*iii*) the recovery rate of low-abundance serum proteins. To follow the reduction in specific peptide signals at different AcCN concentrations, 10 μl human serum was incubated in the presence of 10%, 50%, 60% or 80% AcCN, according to the procedure described above. These concentrations were chosen based on significant signal intensity differences visible in Figure2A. Proteins in the supernatant were trypsin digested and resulting peptides were analysed by MALDI-TOF MS. We focussed on five high-abundance proteins and measured the area under characteristic mass peaks (Fig.3). The comparison of the obtained values clearly shows that the most efficient depletion of high-abundance serum proteins occurs at an AcCN concentration of 60%. Individual depletion rates varied between 81.6% and 100%, resulting in a mean depletion at 60% AcCN of 94.9%.

To evaluate the overall amount of depleted high-abundance proteins, 100 μl serum was incubated with 60% AcCN as described above. After centrifugation, a total amount of 36 μg protein remained in the soluble fraction (not shown). Given a reference interval of 60 to 85 g protein per litre serum, we estimate the depletion rate to be higher than 99%, which is comparable to the depletion rates of individual high-abundance proteins shown in Figure3.

Many low-abundance biomarkers also have a low molecular weight in respect to high concentrated serum proteins, such as albumin and immunoglobulins. The above described AcCN fractionation generally depletes high molecular weight proteins, whereas proteins of low molecular weight remain in solution. Trypsin represents a small protein of 223 amino acids and in addition needs to remain soluble under our partial denaturation protocol, to efficiently cleave the peptide bonds of target proteins. For these two reasons, we used trypsin as a model protein to estimate the recovery rate of low molecular weight proteins. Trypsin of 10 μg was treated with 60% AcCN, centrifuged and the amount of remaining trypsin in the soluble fraction was analysed on SDS-PAGE by densitometry (data not shown). Comparing the signal intensities of 10 μg untreated trypsin with the enzyme amount present in the soluble fraction, we calculated a recovery rate of 97.6%. In addition, our data prove that at an AcCN concentration of 60%, more than 97% of trypsin remains in the soluble fraction and thus most likely is enzymatically active.

The evaluations of our method demonstrate an efficient depletion of high-abundance proteins and a high recovery of small size low-abundance biomarkers. To compare the calculated depletion and recovery rates with more classical techniques, we used immobilized antibodies against human serum albumin. Application of 10 μl serum to an albumin affinity column depleted only about 49.2% of proteins and the recovery of a typical low-abundance protein (growth hormone) was measured to be 75.8% (data not shown). In regard of these data, protein fractionation with 60% AcCN seems to be at least comparable, if not more efficient.

### Size exclusion chromatography of serum proteins

**Figure 4:**
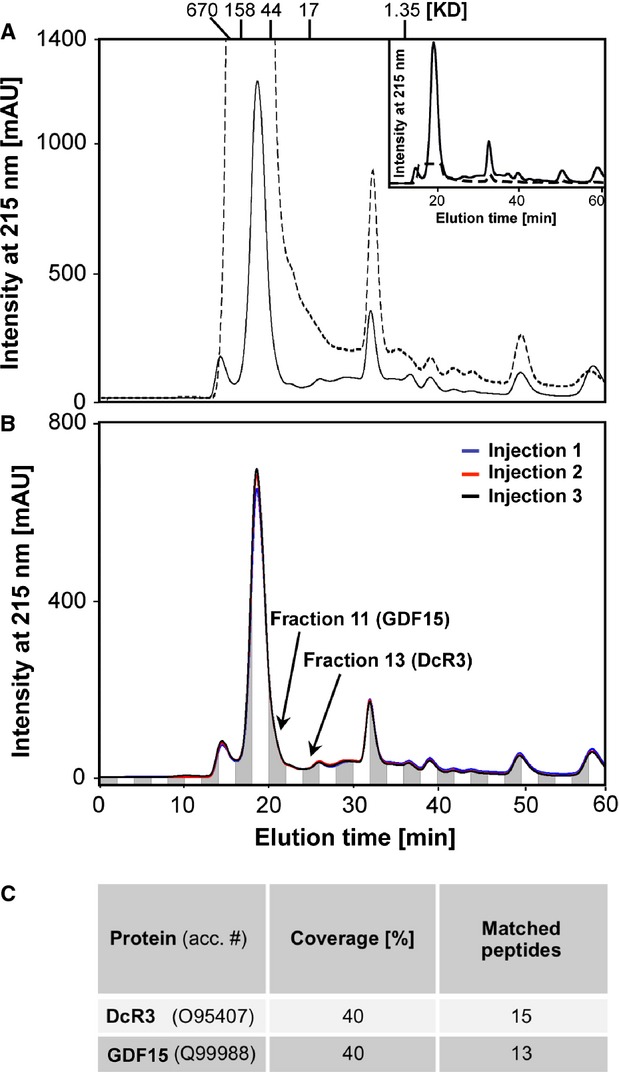
Chromatographic purification of low-abundant serum proteins. (A) Gel filtration of the soluble supernatant of human serum proteins that were partially denatured with 60% AcCN as shown in the white rectangle of Figure2A (solid line). The elution profile of unpurified human serum proteins is shown as dashed line. Eluted proteins were detected at 215 nm to measure the absorbance of peptide bonds. The intensity of elution peaks is shown as milli absorption units (mAU) on the left. Protein masses calculated from a calibration curve are given in kD on top of panel. In the inset, measured intensities representing unpurified human serum proteins (dashed line) were normalized in respect to enriched proteins by a factor of 14.52, based on a 14.52-fold reduction of albumin by our AcCN treatment shown in Figure3. Signals above 2000 mAU (here normalized to 137.74 mAU) were outside the detection range of the gel filtration and thus are clipped. (B) Three separate injections of AcCN treated human serum proteins result in reproducible elution profiles (coloured lines). Fractions 11 and 13 containing GDF15 and DcR3, respectively, are indicated by arrows. (C) Serum proteins present in fraction 11 and 13 were digested with trypsin and resulting peptides were analysed by MALDI-TOF MS. The calculated coverage rates of GDF15 and DcR3 matching peptides identified the two proteins (acc. # - accession number)

To further reduce the complexity of the serum protein composition, we used size exclusion chromatography. Human serum of 100 μl was incubated with 60% AcCN as in Figure2A, the resulting supernatant was analysed by gel filtration and eluted proteins were detected at 215 nm to detect peptide bonds (Fig.4A). Comparison of these chromatograms with the elution profile of untreated human serum proteins (dashed lines in Fig.4A) clearly shows a depletion of high molecular masses, indicating an enrichment of smaller proteins. This can be better seen in the inset of Figure4A, in which the elution profile of untreated human serum proteins was normalized by a factor of 14.52, based on a 14.52-fold reduction in albumin by our AcCN treatment as shown in Figure3A. Furthermore, the comparison of gel filtration chromatograms of replicated injection of AcCN-treated human serum proteins revealed a high reproducibility of our method (Fig.4B). For subsequent analysis, 30 elute fractions were collected over a time period of 60 min., of which fraction 9–24 were subjected to MALDI-TOF MS. The generated spectra identified GDF15 in fraction 11 and DcR3 in fraction 13 (Fig.4B). To verify the identity of DcR3 and GDF15, eluate fractions 11 and 13 were trypsinated and peptide masses obtained by MALDI-TOF MS were matched to predict masses from a theoretical trypsin digest. Coverage rates of about 40% ensured the identity of the two proteins (Fig.4C).

### Selection of fingerprint peptides representing DcR3 and GDF15

To quantify DcR3 and GDF15 in human serum, we combined the above described purification protocol with the quantification concatamer (QconCAT) technology and with IDMS. A ^15^N isotopically labelled QconCAT protein Q1, carrying concatamerized tryptic fingerprint peptides for DcR3 and GDF15 was used as an internal standard for the absolute quantification of the two biomarkers from purified human serum samples by MS.

**Figure 5:**
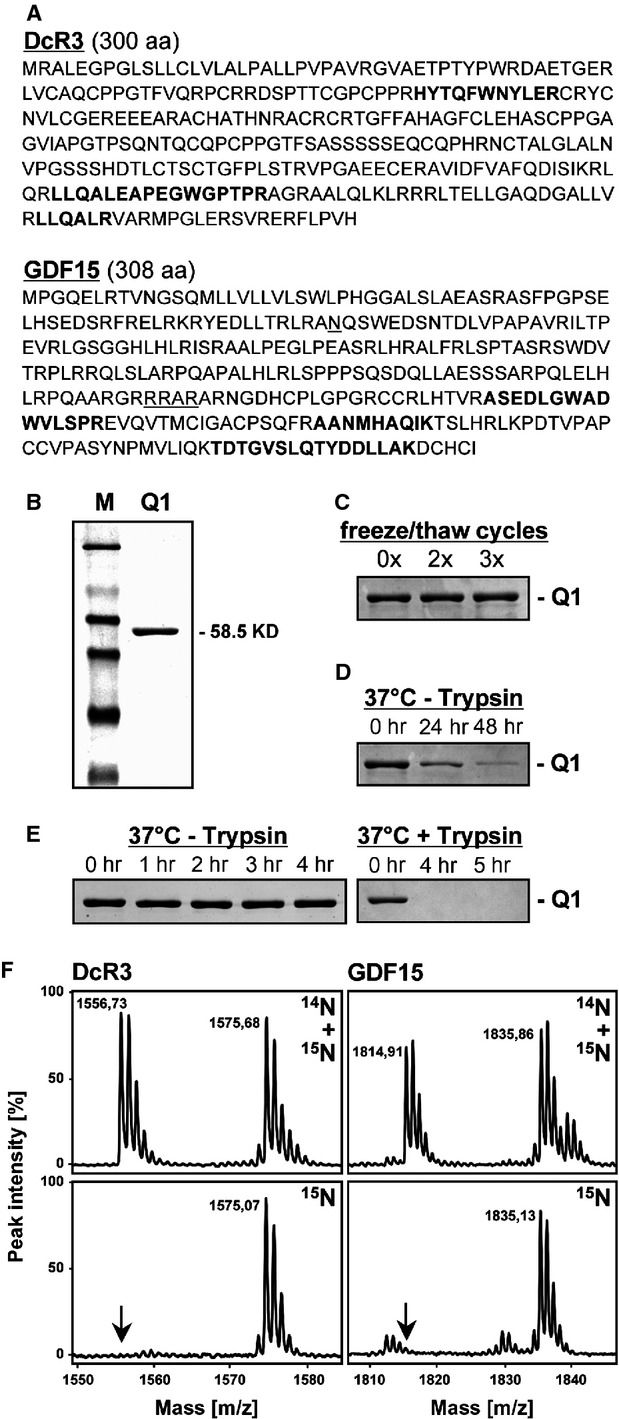
Characterization of the QconCAT Q1 protein. (A) Amino acid sequences of DcR3 and GDF15 indicating localization and primary structure of fingerprint peptides (bold) that were concatamerized in the QConCAT protein Q1. A potentially glycosylated asparagine residue at position 70 and the Furin cleavage site in GDF15 are underlined. (B) Three μg of recombinantly expressed and affinity purified Q1 was analysed on a 12.5% Commassie-stained SDS-PAGE (M – protein marker). Commassie-stained SDS-PAGEs showing the impact of repeated freeze/thaw cycles (C) or incubation at 37°C in the absence (−) or presence (+) of trypsin (D and E) on the stability of Q1. Without proteases, Q1 was stable for up to 4 hrs at 37°C, whereas the addition of trypsin resulted in a complete proteolysis within this time frame, indicating a quantitative liberation of the concatamerized fingerprint peptides. (F) A mixture of unlabelled (^14^N) and isotopically labelled (^15^N) peptides (upper panels), or of labelled peptides (^15^N, lower panels) were analysed by MALDI-TOF MS. Arrows point to the m/z ratio of the unlabelled peptides, indicating a quantitative labelling of Q1. Monoisotopic peaks representing monoprotonated peptides are labelled with their corresponding masses, as listed in Figure7A.

The three fingerprint peptides representing DcR3 or GDF15 are highlighted in the primary sequence of the two proteins (Fig.5A). Peptide sequences were chosen to optimize trypsin digestion efficiency and robust detectability by MS, based on suggestions from van den Broek *et al*., 2013 4. In detail, we tried to realize a preferable peptide length between 8 to 15 amino acids to reduce charge state distribution. Furthermore, proline residues located C-terminal to the cleavage site and the occurrence of two basic amino acids next to each other were avoided because these conditions were described to reduce trypsin digestibility and stability of peptides.

Except one, all peptide sequences representing DcR3 and GDF15 are unique when searched in protein databases for human proteins. Only the sequence LLQALR of DcR3 is also present in other human proteins (Q9ULZ3, Q8IXQ9, Q8WxF8, Q9NVH0, Q93075, Q7Z3E5, Q3MJ16, Q15413). However, these proteins do not contain a trypsin cleavage site (arginine or lysine residue) located in front of the N-terminal leucine and thus will not interfere with the quantification of DcR3.

In contrast to DcR3, GDF15 is extensively post-translationally processed, as reviewed by Mimeault and Batra 2010 23. The major form of the secreted biological active GDF15 is a dimer of the C-terminal 112 amino acid portion of the precursor protein. During biosynthesis, GDF15 may be glycosylated at amino acid 70, which is located N-terminal of the protease cleavage site liberating the C-terminal 112 amino acids. On the basis of this information, we designed all three peptides representing GDF15 within the C-terminal 112 amino acid long region.

### Expression and characterization of the quantification concatamer Q1 protein

To characterize the recombinantly expressed Q1, we analysed (*i*) concentration, purity and size (*ii*) the stability of the protein and (*iii*) the efficiency of metabolic labelling with ^15^N containing amino acids.

1. To reliably determine the Q1 concentration, we used two independent methods. A colorimetric assay and densitometry calculated nearly identical protein concentrations of 50 and 55 μg/ml respectively. As Q1 contains a His-tag fused to its C-terminus, we expected that only completely synthesized proteins were purified by affinity chromatography. Indeed, inspection of Q1 by SDS-PAGE proofed that the protein was of high purity and in agreement with the calculated size (Fig.5B).
2. As the proteolysis of serum proteins by trypsin involves incubation steps at 37°C for several hours, we monitored the stability of synthesized Q1 proteins over various time periods at different temperatures in the absence and presence of trypsin. While freeze/thaw cycles had no visible effect on protein stability (Fig.5C), incubation at 37°C for 24 or 48 hrs without trypsin significantly reduced the Q1 concentration (Fig.5D). In contrast, no degradation of the Q1 protein was visible at 37°C for up to 4 hrs in the absence of the protease (Fig.5E, left panel). This time period was long enough to digest Q1 by trypsin to completion, suggesting a quantitative liberation of the concatamerized fingerprint peptides under these conditions (Fig.5E, right panel).
3. Finally, we analysed the amount of ^15^N incorporated into the recombinantly expressed Q1 protein. Identical amounts of unlabelled or labelled Q1 were individually digested by trypsin and resulting fingerprint peptides were mixed together. As exemplified for one of the three peptides representing DcR3 or GDF15, labelled heavier peptides were easily distinguishable from their unlabelled lighter counterparts by MALDI-TOF MS (Fig.5F, upper panels). Next, we estimated the amount of incorporated ^15^N by analysing labelled peptides only. The spectrograms show a nearly complete absence of the unlabelled lighter peptides, indicating a nearly quantitative labelling efficiency (Fig.5F, lower panels). From this data, we estimated the amount of unlabelled Q1 to be less than 1% and thus did not correct for labelling efficiency in successive quantifications of biomarkers in human serum.

### Visibility of selected fingerprint peptides in ESI-MS

**Figure 6:**
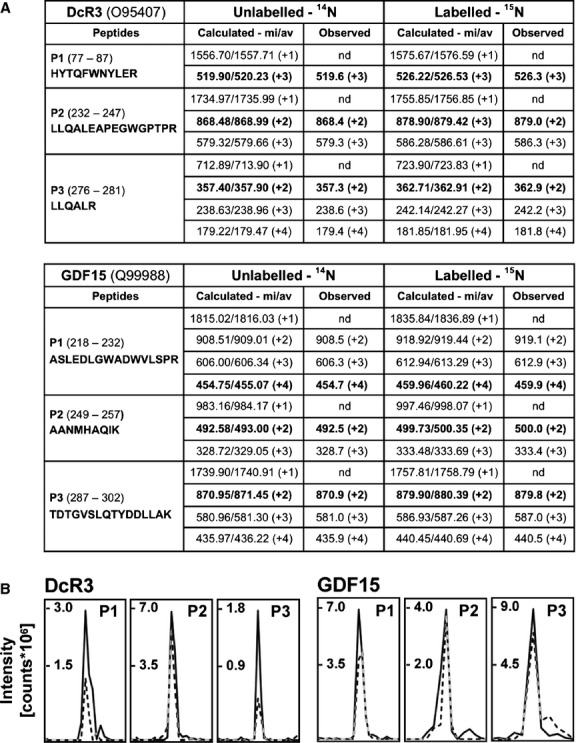
Detection of fingerprint peptides for DcR3 and GDF15 by mass spectrometry. (A) Overview of Q1-derived peptide sequences P1, P2 and P3 and corresponding masses of unlabelled (^14^N) and labelled (^15^N) molecules. Different protonation levels of peptide masses ([M+x H]^x+^) that were detected by ESI MS are indicated in brackets. Monoprotonated peptides were detected by MALDI-TOF MS, only. Peptide protonations used for the absolute quantification of biomarkers are highlighted in bold. (B) A trypsin-digested mixture of 1200 fmol unlabelled and labelled Q1 was analysed by LC-MS/MS. Shown are the extracted ion chromatograms for selected m/z values as specified in bold in (A) that were calculated by MS Digest (unlabelled Q1 – solid line; labelled Q1 – dashed line; av – isotopically averaged mass; mi – monoisotopic mass; nd – not detected by ESI MS).

Because different peptide sequences may generate variable signal intensities in mass spectrometric detection, we tested the visibility of the designed fingerprint peptides by ESI-MS. After trypsination and mixing of unlabelled or ^15^N-labelled Q1 we were able to detect all fingerprint peptides for DcR3 and GDF15 (Fig.6A). Observed masses of di-, tri- and tetra-protonated peptides were in agreement with theoretical predictions, whereas mono-protonated peptides were not detected by ESI MS. Those protonations that represented the most easily detectable signals for each peptide were chosen for successive quantification of DcR3 and GDF15 (Fig.6B and bold numbers in Fig.6A).

### Sensitivity and linear range of the detection technique

**Figure 7:**
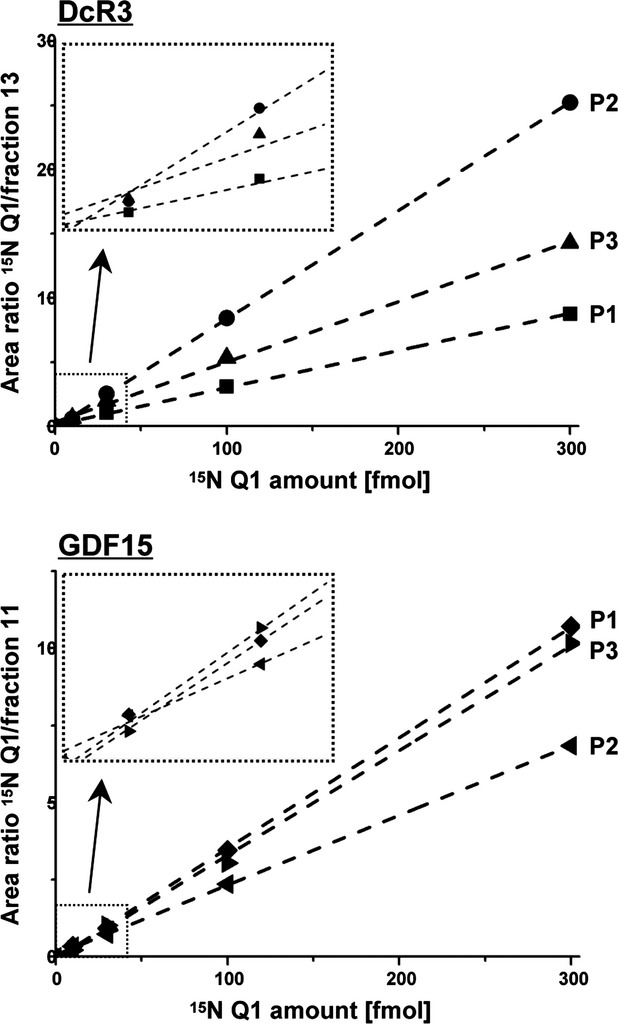
Determination of the linear range of fingerprint peptide detection in the context of human serum. Increasing concentrations of ^15^N-labelled Q1-derived fingerprint peptides P1, P2 or P3 (highlighted in bold in Fig.6) were spiked into gel filtration eluat fractions that contained endogenous DcR3 (fraction 13) or GDF15 (fraction 11, as shown in Fig.3B) and ratios of LC-MS/MS derived areas representing labelled and endogenous peptides were plotted against the amount of labelled peptides. Linear fits are visualized as dashed lines and yielded correlation coefficients between 0.997 and 0.999. The insets enlarge the concentration range between 0 and 40 fmol for better visualization. Fingerprint peptides were analysed by ESI MS as in Figure6.

To determine the sensitivity and linear range of our detection technique, we spiked increasing concentrations of ^15^N-labelled Q1-derived fingerprint peptides into human serum fractions from healthy individuals that contained DcR3 or GDF15 (fraction 13 or 11, see Fig.3B). For every peptide highlighted in bold in Figure6B, the ratio of ESI MS generated peak areas between increasing concentrations of labelled fingerprint peptides and unlabelled, endogenous biomarkers was determined and plotted against the fingerprint peptide concentration (Fig.7). Our data show that we are able to reliably detect as low as 10 to 30 fmol of the fingerprint peptides in the context of human serum. All correlation coefficients were between 0.997 and 0.999, indicating that the relation between peak areas and peptide concentrations is linear in the lower fmol range, which covers physiological concentrations of most biomarkers in human serum.

### Absolute quantification of DcR3 and GDF15 from human serum

**Figure 8:**
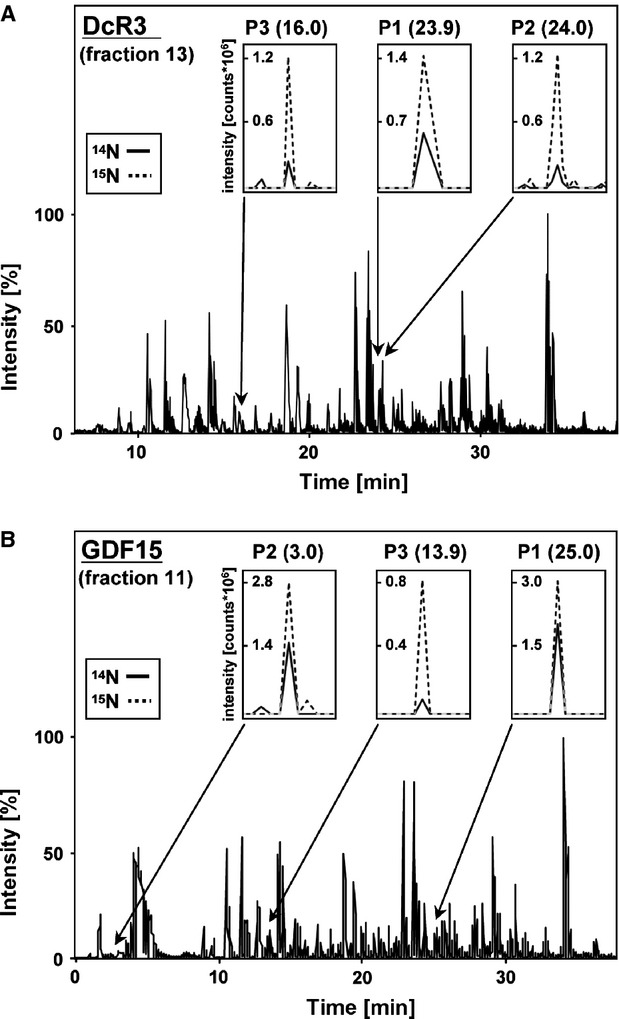
Quantification of biomarkers from human serum using metabolic labelling. (A) Base peak ion chromatogram and extracted ion chromatograms for selected m/z values obtained from a mixture of gel filtration fraction 13 containing DcR3 (see Fig.4B) and ^15^N-labelled fingerprint peptides by ESI MS. The concentration of DcR3 was calculated by dividing the peak areas representing serum proteins by the values of the metabolically labelled internal standard peptides. Peptide retention times shown in brackets are in minutes. (B) Base peak ion chromatogram and extracted ion chromatograms obtained from a mixture of gel filtration fraction 11 containing GDF15 (see Fig.4B) and ^15^N-labelled fingerprint peptides by ESI MS, as described in (A).

Processed eluate fractions 11 and 13 containing tryptic peptides that represent serum levels of GDF15 and DcR3, respectively, were mixed with 50 fmol of ^15^N-labelled and trypsin-digested Q1. For analysis, we used the ESI ion trap MS system in the full-scan modus and generated extract ion chromatograms for masses representing peptides of interest (Fig.8). The amounts of DcR3 and GDF15 were determined by calculating the ratio between the areas of the obtained mass peaks representing DcR3 or GDF15 and the respective values of the ^15^N-labelled Q1-derived fingerprint peptides.

**Figure 9:**
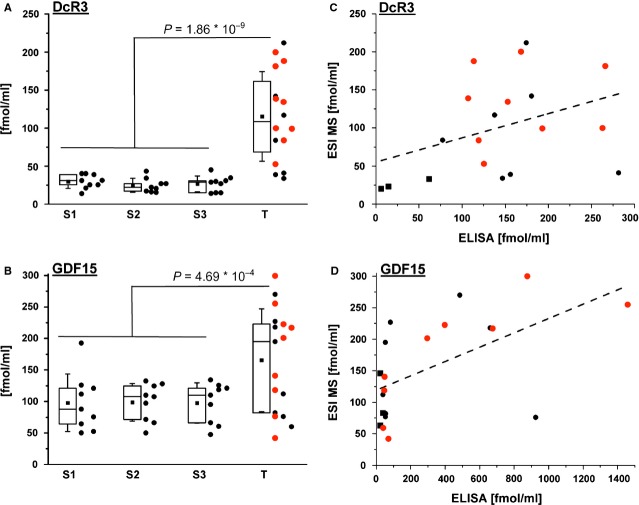
Comparison of DcR3 and GDF15 between sera of healthy and tumour patients. Serum concentrations for DcR3 (A) or GDF15 (B) were calculated by dividing the peak areas representing serum proteins by the values of the metabolically labelled internal standard peptides, as shown in the insets of Figure8. Obtained values are summarized for each of the three control sera of healthy individuals (S1 to S3) and for 16 (A) or 17 (B) sera from tumour patients (T) in box blots. Mean serum levels were comparable between control sera (DcR3: S1 – 29.89 ± 9.12 fmol/ml; S2 – 24.95 ± 9.28 fmol/ml; S3 – 26.87 ± 10.40 fmol/ml; GDF15: S1 – 97.96 ± 45.52 fmol/ml, S2 – 98.66 ± 29.68 fmol/ml, S3 – 97.72 ± 31.90 fmol/ml), but significantly elevated in tumour patients (DcR3: T – 116.94 ± 57.37 fmol/ml; GDF15: T – 164.44 ± 79.31 fmol/ml; all values are ±SD). The vertical extension of the boxes represent lower and upper quartiles, horizontal bars are medians, squares are mean values and whiskers represent standard deviations (±SD). Individual calculated data points are added to the right side of each box. Black or red data points indicate low stage (I or II) or high stage (III or IV) classified tumours. Comparison of DcR3 (C) or GDF15 (D) concentrations obtained by ESI MS and ELISA yielded correlation coefficients of 0.46 (*P* = 0.049) for DcR3 and 0.57 (*P* = 0.009) for GDF15 (squares – control sera; circles – tumour sera). Linear fits are visualized as dashed lines.

For the three control serum samples of healthy individuals, the absolute amount of the biomarkers was analysed using three fingerprint peptides in three independent experiments, resulting in a total of 27 data points for each marker (Fig.9A and B). Obtained mean concentrations were similar between the three healthy sera tested for DcR3 (S1: 29.89 ± 9.12 fmol/ml; S2: 24.95 ± 9.28 fmol/ml; S3: 26.87 ± 10.40 fmol/ml) and for GDF15 (S1 97.96 ± 45.52 fmol/ml, for S2: 98.66 ± 29.68 fmol/ml and for S3: 97.72 ± 31.90 fmol/ml). Comparing the three mean values, we calculated a DcR3 concentration of 27.23 ± 2.49 fmol/ml and a GDF15 concentration of 98.11 ± 0.49 fmol/ml in healthy serum, resembling a variability of 9.14% or 0.5% respectively. In addition to the comparison of the mean values, we also analysed the variability between all 27 individual data points, which resulted in 27.23 ± 9.47 fmol/ml (DcR3) and 98.11 ± 34.96 fmol/ml (GDF15), corresponding to about 35% variability in both cases.

As proof-of-principle, we tested the feasibility of our protocol for the analysis of biomarkers in patient sera. To this end, we defined a collective of 19 colorectal cancer patients. During our studies one patient had no tumour resection and thus his sample was excluded. Of the 18 remaining tumours, nine were classified in stages I or II, whereas the other nine belonged to stages III or IV. Serum concentrations in tumour samples were determined using the P2 fingerprint peptide for DcR3 and P1 for GDF15. Analysing all patient sera, mean values were calculated to 116.94 ± 57.37 fmol/ml for DcR3 and 164.44 ± 79.31 fmol/ml for GDF15 (Fig.9A and B). For DcR3, eight of nine tumours that were classified in stages III or IV (highlighted in red in Fig.9A) also showed biomarker concentrations above the controls, whereas for GDF15 this was just the case for five samples (Fig.9B). For both biomarkers, about half of the samples representing tumour stages I or II (black data points in Fig.9A and B) correlated with low biomarker concentrations. Correlating histopathological grading with the measured biomarker concentrations resulted in a similar picture (data not shown).

**Figure 10:**
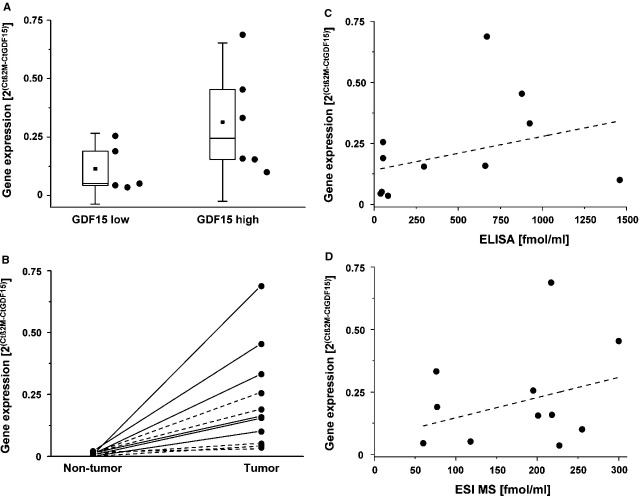
Comparison of GDF15 sera concentrations with transcript levels in formalin-fixed and paraffin-embedded sections of colon tumour samples by qPCR. (A) qPCR data of samples containing low or high GDF15 serum concentrations, as shown in Figure9D. Box blots and data points are assembled as in Figure9. The housekeeping gene β2-microglobulin (β2M) was used for normalization. (B) GDF15 transcript levels were compared between tumour central regions (tumour) and normal regions (non-tumour) of the same patient. Pairs of data points representing tumour and normal areas of one section are connected by lines (GDF15 low – dashed lines; GDF15 high – solid lines). (C and D) Comparison of GDF15 transcript levels with sera concentrations obtained by ELISA or ESI MS. Dashed lines represent linear fits of the data points.

Next, we compared the DcR3 and GDF15 serum concentrations form our ESI MS method with values obtained by an independent technique (ELISA), as well as with the gene expression of the two biomarkers in tumour sections of the same patients, measured by qPCR. Correlations between ESI MS and ELISA calculated Spearman’s correlation coefficients to be 0.46 (*P* = 0.049) for DcR3 and 0.57 (*P* = 0.009) for GDF15 (Fig.9C and D). To compare sera concentrations obtained by ESI MS and ELISA with transcript levels, we selected six or five formalin-fixed and paraffin-embedded sections containing tumours from patients with high or low GDF15 serum levels, respectively, based on our data presented in Figure9D. Comparable to the measured GDF15 protein levels in serum, also the GDF15 transcript levels in tumour sections showed a clear distinction between both patient groups (Fig.10A). The obtained qPCR values were specific, as GDF15 transcript levels from normal regions showed lower expression levels than those from tumour cells (Fig.10B). Finally, we correlated the obtained GDF15 transcript levels with the previously obtained GDF15 serum levels (Fig.10C and D). Because of the low sample number, both correlations did not reach statistical significance (Fig.10C: *R* = 0.482, *P* = 0.138; Fig.10D: *R* = 0.294, *P* = 0.381). However, patients with low/high GDF15 serum concentrations had a clear tendency to show also a low/high GDF15 expression in tumour cells, as indicated by the positive slope of the fitted lines.

## Discussion

Biomarkers are important tools in clinical prognosis, diagnosis and therapy monitoring 2. Here, we present a new method for the absolute quantification of small-sized and low concentrated biomarkers from 100 μl human serum. Our experimental approach combines the depletion of high-abundance serum proteins by acetonitrile (AcCN) treatment, the enrichment of low-abundance serum proteins by size exclusion chromatography, the identification of biomarkers *via* peptide matches and the quantification of biomarkers using fingerprint peptides of known concentrations that serve as metabolically labelled internal standards.

The absolute quantification of proteins requires an efficient purification and a quantitative proteolysis of biomarkers. To this end, we optimized the partial denaturation of high-abundance serum proteins by AcCN in the presence of different salt concentrations. Under some experimental conditions, the transition from soluble to insoluble proteins occurred over a broad AcCN range, as evident *e.g*. for the disappearance of soluble serum proteins in Figure2A and C between 40% and 60% AcCN. In other cases, we observed a transition between soluble to insoluble proteins within a 5% difference of AcCN concentration only, *e.g*. between 50% and 55% AcCN for soluble proteins in Figure2A, or between 40% and 45% for the precipitate in Figure2C. We speculate that individual serum proteins possess distinct sensibilities to variations in salt and/or AcCN concentrations, which might result in very similar, or in more diverse solubility products, depending on the experimental conditions used. While a clear correlation between increasing AcCN concentrations and a reduced solubility of serum proteins was evident under all experimental conditions, a correlation between increasing salt concentration and protein solubility was not obvious.

Partial denaturation of serum proteins by 60% AcCN resulted in depletion rates of high-abundance proteins between 81.6% and 100% (mean of 94.9%), which is roughly equivalent to a 94.9-fold enrichment of proteins remaining in the soluble fraction. The recovery rate of proteins present in this soluble fraction was determined to be 97.6%. From these data, we can estimate an overall 92.62-fold enrichment (97.6% recovery of a 94.9-fold enrichment) for small- and low-abundant serum proteins during our purification procedure. Compared to established purification techniques, such as albumin affinity columns that in our hands yielded depletion and recovery rates of about 49% and 76%, respectively, the newly designed experimental approach seems to be well suited for biomarker quantification.

Besides the enrichment and purification of biomarkers, the use of AcCN has further advantages for protein quantification. The partial denaturation of serum proteins induced by AcCN treatment reduces protein–protein interactions, *e.g*. between albumin and low concentrated serum proteins. As a result, the amount of soluble and freely accessible biomarkers is increased and represents more accurately the real protein concentration in human serum. In addition, the partial unfolding of protein structures induced by AcCN ensures a complete digest of biomarkers by trypsin, a prerequisite for their reliable detection by MS.

In the literature, several biomarkers are described as promising tumour markers in colorectal cancer, including DcR3, GDF15, M2-PK, PSME3 and TIMP-1 13. Originally, we designed fingerprint peptides for all five biomarkers. Our preliminary tests revealed that fingerprint peptides representing DcR3 and GDF15 showed the most reliable and reproducible results in MS. Furthermore, the identity of DcR3 and GDF15 was ensured by 15 or 13 matching peptides, respectively, resulting in both cases in a coverage of 40%. In contrast, M2-PK, PSME3 and TIMP-1 showed coverage rates of 12%, 16% or 32%, only. Therefore, in this study we concentrated on DcR3 and GDF15. Both proteins are discussed in the literature as important mediators of cell survival of tumour cells and were suggested to be used as biomarkers for cancer patients suffering from various tumours, including colorectal, pancreatic, prostate, ovarian cancer and melanoma 14,15,24–28.

DcR3 is a soluble and secreted member of the tumour necrosis factor receptor family. The receptor antagonized the apoptotic activity of the death receptor 3 and thus protects tumour cells from cell death 29. This ‘reverse signalling’ is mainly achieved by a competition between the death receptor 3 and DcR3 for specific ligands, including the vascular endothelial growth inhibitor, the Fas ligand FasL and the cytokine LIGHT 30. In addition, DcR3 may suppress the immune system 31. Thus, the receptor is discussed in the literature as important mediator of cell survival in cancer and indeed, DcR3 is highly expressed in and secreted from various tumours, *e.g*. in colorectal and ovarian cancer 14,24.

Under normal physiological conditions DcR3 is low concentrated in human serum. In contrast, about 56% of 146 cancer patients tested showed elevated DcR3 levels in their blood, with large variations depending on the tumour type 32. Analysing sera of 39 healthy individuals by ELISA resulted in a mean concentration of 0.56 ± 0.52 ng/ml (16.97 ± 15.76 fmol/ml), where in 59 tumour patients suffering from diverse cancer types DcR3 amounts were about four times higher (2.3 ± 1.6 ng/ml; corresponding to 69.70 ± 48.48 fmol/ml) 33. Using three different fingerprint peptide sequences as internal standard, our protocol yielded DcR3 concentrations of 29.89 ± 9.12 fmol, 24.95 ± 9.28 fmol and 26.87 ± 10.40 fmol/ml in three different control sera. In contrast, we found DcR3 values significantly elevated with a mean of 116.94 ± 57.37 fmol/ml, in agreement with our ELISA measurements (mean: 168.53 ± 58.30 fmol/ml) and with the published data described above.

Growth/differentiation factor 15 is a member of the transforming growth factor beta family, involved in cellular stress and immune responses 23. Furthermore, GDF15 has protective and anti-apoptotic characteristics. GDF15 expression was found to be elevated in tumours and in serum samples from patients suffering from and various cancer types, including pancreatic, colorectal and prostate cancer and melanoma and may cause resistance to chemotherapeutics 15,25–28. Therefore, GDF15 is discussed in the literature as a biomarker for various cancer types. As GDF15 is the only known cytokine regulated by the tumour suppressor protein p53, it can serve as a biomarker for p53 activity. However, other transcription factors, such as the nuclear factor kB can regulate GDF15 expression, as well 23.

Elevated GDF15 levels were correlated with poor patient survival and causative molecular mechanisms including a facilitation of proliferation, migration and invasion of cancer cells were suggested 28,34. Thus, GDF15 is discussed as a biomarker for cancer patients. However, in early stages of the disease, GDF15 can also act as a tumour suppressor and play protective roles, *e.g. via* inhibiting tumour growth and by pro-apoptotic activities.

Two publications of the same group analysed GDF15 serum concentrations in pancreatic cancer patients by ELISA 26,35. In healthy individuals, mean GDF15 levels were 0.55 ± 0.26 ng/ml (43.64 ± 20.94 fmol/ml) or 0.76 ± 0.41 ng/ml (60.67 ± 32.93 fmol/ml) respectively. In contrast, in pancreatic cancer patients GDF15 concentrations were about four to sevenfold higher (80 patients: 2.43 ± 2.32 ng/ml; corresponding to 194.24 ± 185.45 fmol/ml), or (50 patients: 5.39 ± 3.72 ng/ml; equivalent to 430.86 ± 297.36 fmol/ml). Another study reports elevated GDF15 levels in 58 colorectal cancer patients (0.78 ± 0.49 ng/ml; 62.59 ± 39.25 fmol/ml)), in respect to the mean concentration in 260 healthy individuals (0.50 ± 0.21 ng/ml; 39.57 ± 16.79 fmol/ml) 15. Similar to the reported data, we determined mean GDF15 concentrations in three control sera to be 97.96 ± 45.52 fmol/ml, 98.66 ± 29.68 fmol/ml and 97.72 ± 31.90 fmol/ml, whereas we found significantly elevated protein levels by ESI MS (mean: 164.44 ± 79.31 fmol/ml) and by ELISA measurements (mean: 378.1 ± 411.78 fmol/ml). The calculated standard deviation of our MS measurements (±79.31 fmol/ml) represents 48.2% of the mean GDF15 serum concentration, which is significantly better than the error produced by our ELISA (108.9%), or the variability in the ELISA of the above mentioned 58 colorectal cancer patients (62.7%).

It has been reported that variations in ELISA measurements of serum proteins can result in concentration differences up to 250% within identical serum samples using the same calibration standards, depending on the immunoassay used 36. Especially immunoassays using monoclonal antibodies are vulnerable to differential recognition of molecular variants depending on the unique epitope specificity of the antibody used. Polyclonal assays are more robust in this regard because of ‘epitope averaging’ among the wide spectrum of epitope specificities present in the antibody population. Therefore the standardization of immunoassays is an inherent necessity.

As an alternative, MS offers an unbiased technique for the absolute protein quantification at the fmol level with a good reproducibility. Besides the fact that the generation of highly specific antibodies is not a trivial task and often more expensive than the synthesis of fingerprint peptides, MS techniques can offer a more linear sensitivity than antibody-based approaches, especially at low concentrations. In Figure9D *e.g*. ELISA shows a relative poor distinction between the GDF15 concentrations of 12 samples (20–90 fmol/ml), whereas the ESI MS technique separated the same samples much better (concentrations between 25 and 250 fmol/ml.) A further advantage of the MS technique is the relatively easy realization of multiplexing strategies, with simultaneous detection of different biomarkers, or the detection of several products originating from the same precursor ion (multiple reaction monitoring) 37,38. However, rarely in the literature protein quantification was performed with more than one fingerprint peptide as internal standard for one biomarker 39. Here, we show that using multiple fingerprint peptides for the protein of interest can generate highly reproducible results. Comparing the mean values calculated for each individual control serum, we estimated the reproducibility of our technique by calculating a variability of 9.14% for DcR3 and of 0.5% for GDF15, based on the obtained standard deviations. This is comparable or even better than an accuracy of below 10% discussed in the literature for protein quantification by metabolic labelling techniques 40. However, unlike comparing the mean values, a comparison of all 27 individually measured DcR3 and GDF15 concentrations showed a variability of about 35%. This number might potentially be reduced by an automatization of the presented protocol.

In summary, we developed a new experimental approach for the reliable quantification of low-abundance proteins such as tumour markers from 100 μl human serum. Our technique shows a higher resolution at low biomarker concentrations than ELISA and offers the possibility of designing multiplexed measurements. While we principally demonstrate the feasibility of our protocol for the analysis of biomarkers in human serum, future analysis will be needed to evaluate the prognostic value of DcR3 and GDF15 for colon cancer patients using large patient cohorts.

## Supporting Information

**Table S1** Patient and tumour characteristics included in the DcR3 and GDF15 sera analysis (*n* = 19; NA, not applicable; ^1^no tumour resection, no neoadjuvant therapy for one patient; ^2^Classification missing for one patient).
