# Supplementary material for: Absolute quantification of DcR3 and GDF15 from human serum by LC-ESI MS
Source: J Cell Mol Med. 2015 Mar 30;19(7):1656–71. doi: 10.1111/jcmm.12540 (PMC4511363; doi:10.1111/jcmm.12540)
Supplement: Supplementary file 1 [file jcmm0019-1656-sd1.docx]

**Supplemental Table 1.** Patient and Tumor characteristics included in the DcR3 and GDF15 sera analysis (n=19; NA, not applicable; ^1^no tumor resection, no neoadjuvant therapy for one patient; ^2^Classification missing for one patient)

| **Characteristics** | **Value (n = 19)** | **Relative percentage** |
| --- | --- | --- |
| Male:female ratio | 10:9 = 1.1 | NA |
| Median/range age (years) | 56.8/22-87 | NA |
| Tumor site |  |  |
| Right colon | 5 | 26.3 |
| Transverse colon and flexures | 4 | 21.1 |
| Left colon | 5 | 26.3 |
| Rectum | 5 | 26.3 |
| Pathological stage (UICC 2009)^1^ |  |  |
| I | 5 | 26.3 |
| II | 4 | 21.1 |
| III | 2 | 10.6 |
| IV | 7 | 36.8 |
| pT category^2^ |  |  |
| pT2 | 6 | 31.6 |
| pT3 | 8 | 42.1 |
| pT4 | 4 | 21.1 |
| pN category^2^ |  |  |
| pN0 | 9 | 47.4 |
| pN1 | 4 | 21.0 |
| pN2 | 5 | 26.3 |
| Distant metastases |  |  |
| M0 | 11 | 57.9 |
| M1 | 8 | 42.1 |
| Histopathological grading^2^ |  |  |
| Low grade (G_1_/G_2_) | 12 | 63.2 |
| High grade (G_3_/G_4_) | 6 | 31.6 |
| Lymphatic venous invasion^2^ |  |  |
| L0 | 13 | 68.4 |
| L1 | 5 | 26.3 |
| Venous invasion^2^ |  |  |
| V0 | 17 | 89.5 |
| V1 | 1 | 5.3 |
| Perineural invasion^2^ |  |  |
| no | 16 | 84.2 |
| yes | 2 | 10.5 |
| Stroma fibrosis^2^ |  |  |
| no | 9 | 47.4 |
| yes | 9 | 47.4 |
| Emergency presentation |  |  |
| no | 19 | 100 |
| yes | 0 | 0 |
| R classification |  |  |
| R0 | 12 | 63.2 |
| R1 | 1 | 5.3 |
| R2 | 6 | 31.6 |
| Radiation therapy (postoperative or alone) |  |  |
| no | 16 | 84.2 |
| yes | 3 | 15.8 |
| Chemotherapy (postoperative or alone) |  |  |
| no | 10 | 52.6 |
| yes (adjuvant) | 4 | 21.1 |
| yes (palliative) | 5 | 26.3 |
